# Case Report: Novel Arylsulfatase A (*ARSA*) Gene Mutations in a Patient With Adult-Onset Metachromatic Leukodystrophy Misdiagnosed as Multiple Sclerosis

**DOI:** 10.3389/fneur.2020.576881

**Published:** 2021-01-11

**Authors:** Lulu Xu, Meixiang Zhong, Yajuan Wang, Zhihong Wang, Jie Song, Jing Zhao, Hongyun Yu, Zhencui Yang, Wenjing Yan, Xueping Zheng

**Affiliations:** ^1^Department of Geriatric Medicine, The Affiliated Hospital of Qingdao University, Qingdao, China; ^2^Department of Geriatric Medicine, The Qingdao Eighth People's Hospital, Qingdao, China; ^3^Department of Clinical Laboratory, The Affiliated Hospital of Qingdao University, Qingdao, China; ^4^Department of Neurology, The Affiliated Hospital of Qingdao University, Qingdao, China

**Keywords:** visual dysfunction, deletion mutation, arylsulphatase A, adult onset, metachromatic leukodystrophy

## Abstract

Metachromatic leukodystrophy (MLD) is an autosomal recessive hereditary disorder characterized by the accumulation of sulfatide in the central and peripheral nervous systems. Herein, we present the case of an adult patient with MLD who had mild cognitive and psychiatric dysfunctions and severe vision disturbance, who was initially misdiagnosed as multiple sclerosis. Through genetic screening, this patient was later identified to have a full deletion of exon 4 and the novel p.P220L mutation in the arylsulfatase A (*ARSA*) gene. These mutations are reported for the first time in MLD. These data will help to update the mutation profiles of patients with MLD.

## Introduction

Metachromatic leukodystrophy (MLD) is an autosomal recessive hereditary disorder characterized by the accumulation of sulfatide in the white matter of the central nervous system (CNS) and peripheral nervous system (PNS) as well as in other non-neural tissues (1). It is caused by a deficiency of the sulfatide-degrading lysosomal enzyme arylsulfatase A (ARSA), which leads to progressive demyelination that causes a variety of neurological symptoms (2). Based on the age at onset and disease severity, MLD is classified into at least three variants: late infantile, juvenile, and adult. The late-infantile and juvenile variants comprise a large part of all MLD cases (3). The adult-onset phenotype, in which symptoms appear after 16 years of age, is quite rare and usually presents with cognitive dysfunction, psychiatric disturbances, motor symptoms, or peripheral polyneuropathy (4). Clinically, MLD is challenging to diagnose. Magnetic resonance imaging (MRI) often shows symmetric periventricular white matter involvement sparing the arcuate (U-)fibers, without any contrast enhancement (5). However, no specific imaging characteristics have been recognized. Usually, it may be misdiagnosed as schizophrenia, bipolar disorder, Alzheimer's disease, Pick's disease, or even multiple sclerosis (6, 7). Therefore, a screening assay of *ARSA* gene mutations is helpful to confirm a diagnosis of adult-onset MLD.

The *ARSA* gene is located on the q arm of chromosome 22 and has eight exons comprising a 1.5-kb coding sequence. Several mutations of the *ARSA* gene that cause MLD have been identified. Patients with adult-onset MLD are usually heterozygous for alleles, expressing low residual amounts of enzyme activity; in contrast, late-infantile MLD patients with homozygous alleles often have complete loss of enzyme activity. Obviously, the residual enzyme activity is one of the determinants of clinical severity and age at onset. More than 250 mutations causing MLD have been reported in different populations (8); however, genetically, only three defective alleles are frequently noted: a splice donor-site mutation at the exon 2/intron 2 border, a missense mutation causing a p.P246L substitution, and a p.I179S substitution. All types of mutations, including deletions, insertions, splice site mutations, and missense mutations, have been reported. Missense mutations are the most frequent type of defect in the *ARSA* gene; however, MLD is a rare disorder, especially the adult-onset subtype, which is usually underdiagnosed or misdiagnosed.

The diagnosis of MLD is based on ARSA activity in leukocytes or fibroblasts and sulfatide excretion in the urine. However, molecular diagnosis of MLD is more accurate than conventional methods, and the corresponding samples are more stable than those used for determining enzyme activity (9). Low ARSA activities have also been reported in healthy individuals who are carriers of a pseudodeficiency allele but without clinical signs of MLD (10). The pseudodeficiency allele, which has a prevalence of 10–20% in the general population, further complicates the clinical diagnosis when an enzymatic assay is used (11). This allele causes reduced enzymatic activity, which confounds the biochemical determination of a pseudodeficient state, carrier, and disease (12). Although the pseudodeficiency allele can result in reduced enzymatic activity, it has no correlation with the disease state (13). Therefore, the measurement of ARSA activity was previously limited to providing an accurate diagnosis; however, genetic screening eliminates the uncertainty inherent to enzymatic analysis.

## Case Report

A 41-year-old woman, a cardiology physician, presented to the ophthalmology clinic in July 2014 with a 3-month history of binocular blurred vision and pain with eye movement. The patient's visual acuity continued to decline and was accompanied by double-eyeball swelling and pain. The pain was recurrent, lasting ~2 h per episode, which was further aggravated by eye activity or fatigue and relieved after rest. Her binocular vision decreased from 1.5 to 0.15 in the right eye and was 0.3 in the left eye; however, her visual field, color perception, and ocular fundus examination findings were normal at presentation. After carefully reviewing her medical history, we found that she had been diagnosed with depression due to hypothymia and slow movement in another hospital, before visiting our hospital. Although she received antidepressant treatment, her symptoms continued to progress. Her Self-Rating Depression Scale and Hamilton Depression Rating Scale scores were 64 and 28, respectively. Furthermore, her husband remembered that she had presented with mild learning and concentration impairment when preparing for a doctor's promotion test half a year ago. Because of her worsening vision and psychomotor deterioration, she was referred to our hospital. There was no relevant family history; both her parents were alive, and her younger brother was healthy. At admission, our patient was oriented in time, place, and person/identity. Her thought process was slow, and her behavior was disorganized. The Montreal Cognitive Assessment and Mini-mental State Examination scale scores were 16 and 21, respectively. The patient did not show the Marcus Gunn sign and inflammation of the optic nerve. There were no other positive signs in the neurological examination.

**Figure 1:**
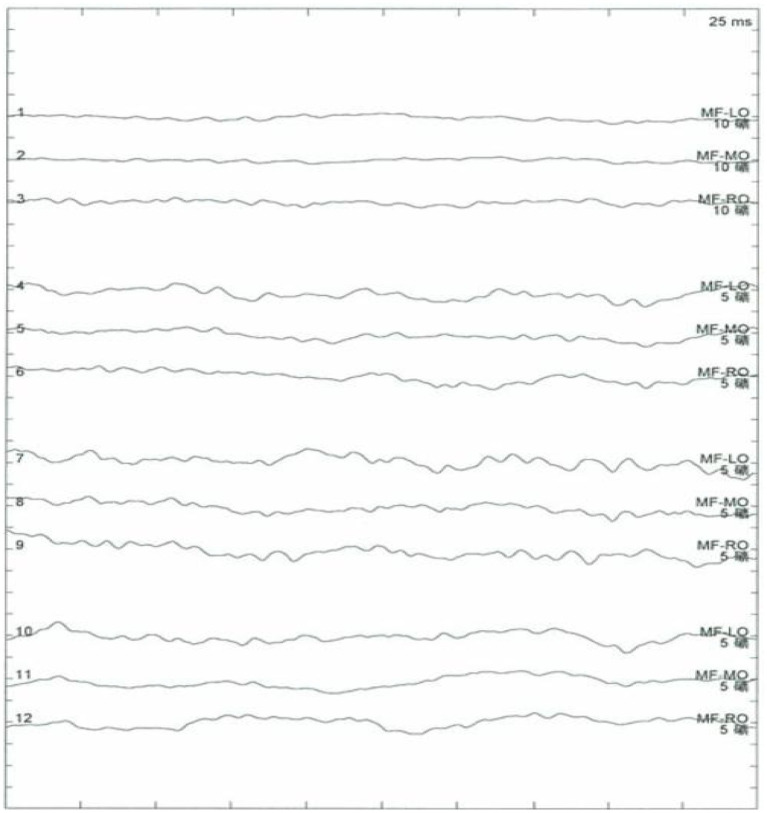
No recordable visual evoked potentials (VEPs; N75, P100, and N145) and VEP waveforms are seen.

**Figure 2:**
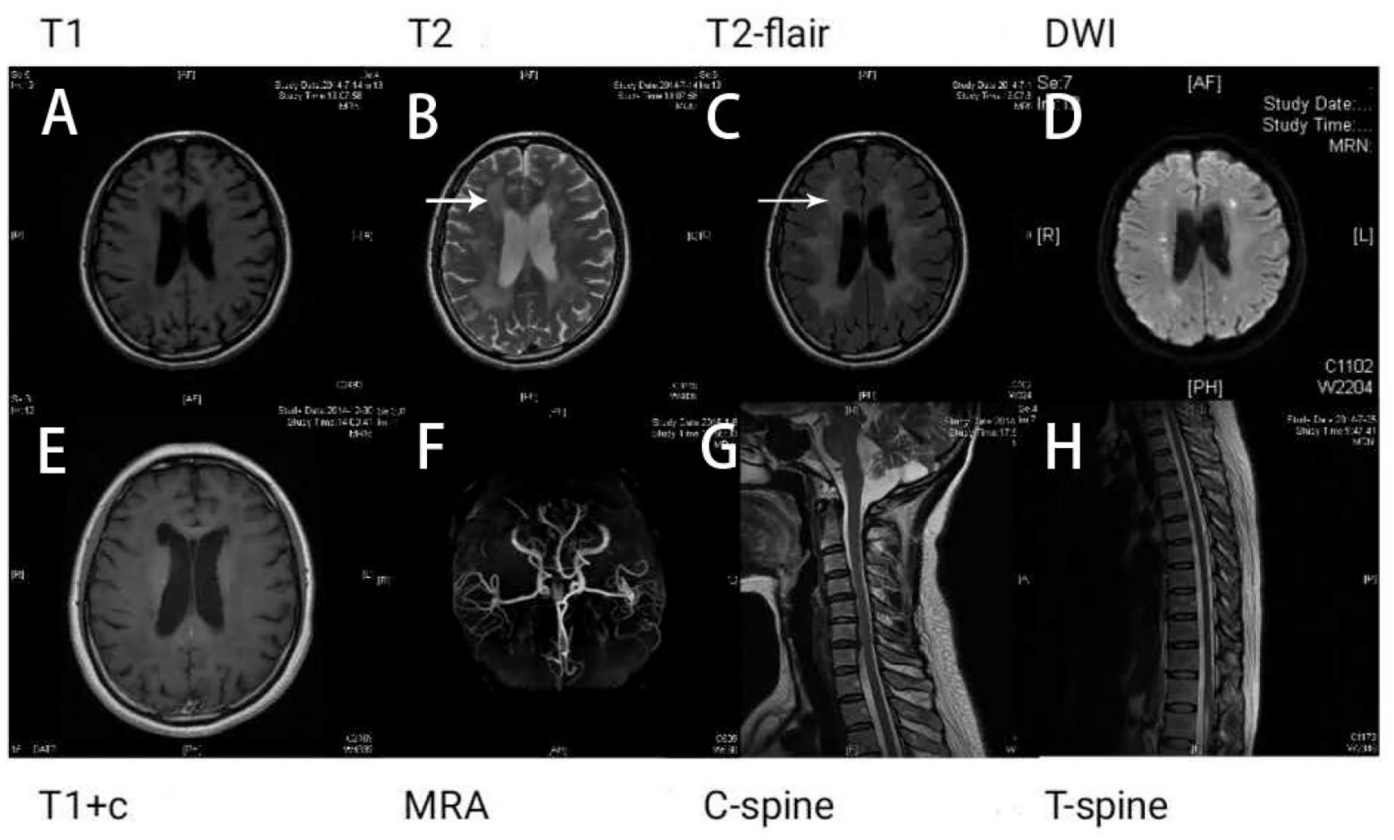
Brain magnetic resonance imaging (MRI) shows white matter lesions. “Tigroid” or “leopard skin” lesions were present in T2-weighted **(B)** and T2-weighted fluid-attenuated inversion recovery (FLAIR) sequences (white arrow) **(C)**. These lesions appear dark in a T1-weighted sequence **(A)**. Bright lesions are present in a diffusion-weighted imaging (DWI) sequence **(D)**. The lesions show no contrast enhancement **(E)**. The patient's magnetic resonance angiography (MRA) **(F)**, cervical **(G)**, and thoracic spinal cord images **(H)** appear normal.

Results of the following studies were normal: ANA, ACA, ANCA, TGAB, ATPO, and dsDNA. The results of routine testing for syphilis and HIV serology were negative. Visual evoked potential (VEP) waveforms (N75, P100, and N145) were undetectable in our patient (Figure 1). Electroencephalography showed abnormal bilateral synchronous slow activities. Protein levels (1.094 g/L) in the cerebrospinal fluid (CSF) were elevated, but the immunoglobulin G (IgG) index and white blood cell count were normal. Her brain MRI showed a diffuse white matter hyperintensity in the bilateral *centrum semiovale* area and bilateral radiating crown area on T2-weighted and T2-weighted fluid-attenuated inversion recovery sequences, demonstrating a “tigroid” or “leopard-skin” demyelination pattern. Cervical and thoracic MRIs were normal (Figure 2).

Based on the results of both clinical and laboratory tests, multiple sclerosis was considered, and treatment with a high dose of methylprednisolone (1,000 mg/day for 5 days) was initiated. After treatment, mild improvement of vision acuity was reported by the patient, but ophthalmologic examination results were unchanged. Results from a second lumbar puncture still revealed elevated proteins (1.005 g/L) and a normal IgG index (0.611) in the CSF.

**Figure 3:**
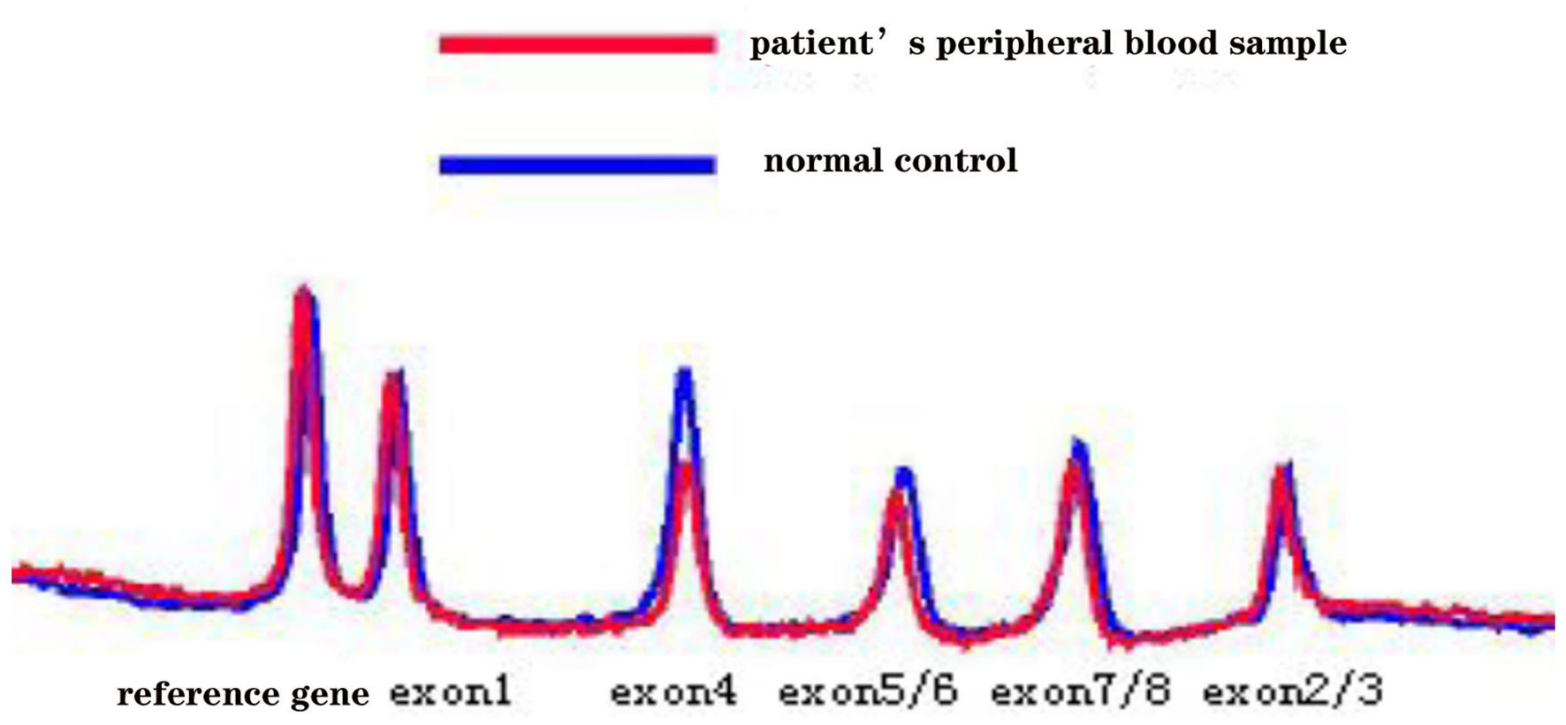
The denaturing high-performance liquid chromatography analysis results show a full deletion of exon 4 in the *ARSA* gene.

**Figure 4:**
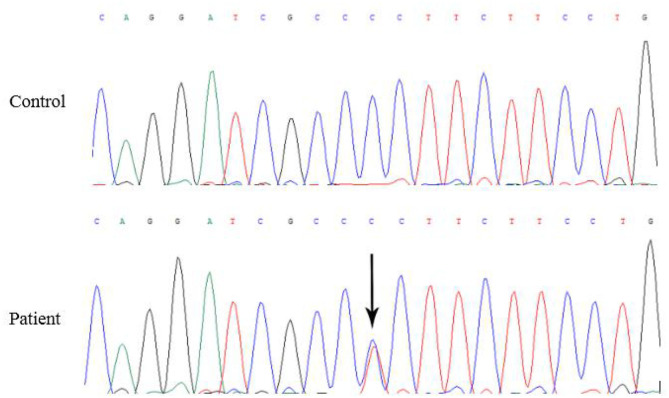
The black arrow shows c.659C>T, a novel Pro220Leu mutation in exon 3 of the *ARSA* gene.

Five months later, the patient's condition continued to progress slowly, and she visited another hospital. The same diagnosis of multiple sclerosis was considered, and plasma exchange was initiated this time. However, little effect was reported by the patient, as her blurred vision improved only mildly. Moreover, 20 days later, the patient developed weakness in her lower extremities, precluding her from walking by herself. She was again admitted to our hospital in December 2014. The neurological examination revealed spastic paralysis of the two lower limbs. Her muscle strength (manual muscle testing using Medical Research Council grading) was grade 3 in her left leg and grade 4 in her right leg. The knee tendon reflexes were brisk, and both Babinski and Rossolimo signs were positive. At this time, the level of proteins in her CSF were elevated, at 1.082 g/L, and the IgG index was 0.723 (normal <0.8). Brain MRI revealed diffuse and symmetric white matter hyperintensity without significant differences with that performed half a year ago. Brain atrophy was conspicuous, showing enlarged ventricles and *corpus callosum* atrophy. Enhancement was not prominent, and magnetic resonance angiography was normal. No waveforms were detected in the VEP test, as before. However, at this time, multiple sclerosis was no longer considered, and MLD was suspected. Tests for sulfatase enzyme activity and urinary sulfatide excretion were not available in our hospital, and gene testing was conducted for this patient. Gene screening showed a full deletion of exon 4 (Figure 3), a novel p.P220L mutation (Figure 4), and a known mutation, p.T393S, in the *ARSA* gene.

## Discussion

Mental deterioration and behavioral abnormalities are the most common symptoms of MLD as described in the literature. Before the examination of brain MRI, ARSA activity, sulfatides, or molecular diagnosis, patients are often misdiagnosed with schizophrenia, affective disorders, or personality disorders (6). Our patient was initially misdiagnosed with multiple sclerosis in two different hospitals because of visual disturbances and periventricular “demyelination.” Her neurological deterioration had been progressing since onset; consequently, primary progressive multiple sclerosis was considered at her second admission. Adult-onset MLD has been reported to mimic primary progressive multiple sclerosis; in previous reports, all patients with adult-onset MLD were misdiagnosed mainly due to a progressive clinical course and findings of periventricular white matter hyperintensity (6, 7). However, none of the previously reported patients had the more common clinical characteristics of multiple sclerosis, such as limb paralysis or numbness. The main complaint of our patient was vision disturbance, which is not a common presentation in adult-onset MLD. Dementia and other psychiatric symptoms have been usually reported as the initial presentations of adult-onset MLD, which were not the chief complaints of our patient. Therefore, MLD was not considered initially.

The lesions seen in the brain MRI are different between multiple sclerosis and MLD. The characteristic demyelinating lesions of multiple sclerosis are located in the periventricular, juxtacortical, infratentorial, and spinal cord locations (14). In contrast, a diagnosis of MLD is considered when the brain MRI shows predominantly symmetric periventricular and subcortical lesions in the temporal, occipital, and frontal regions, sparing the U-fibers. Furthermore, the ventricular system is also symmetrically enlarged, and signs of corpus callosum atrophy are also observed in MLD (14). Our patient had diffuse bilateral symmetrical periventricular lesions with no obvious demyelinating lesions on the brainstem or spinal cord, which is not consistent with multiple sclerosis. According to our own experience, the onset of visual impairment in this patient was relatively slow, which was a gradual process of aggravation. Treatment with high-dose methylprednisolone was not effective for our patient. Moreover, her previous history included cognitive and psychiatric problems, such as attention difficulty and depression, which had been present for at least 1 year. Therefore, the diagnosis of a white matter disease other than multiple sclerosis, especially one with a metabolic origin, such as MLD, was considered.

There is much disagreement in the literature regarding the incidence of common symptoms in adult-onset MLD, which makes its diagnosis difficult and complicated. Hyde et al. (10) stated that 53% of patients with adult-onset MLD present with psychosis as the initial manifestation. However, Hageman et al. (11) suggested that psychosis was a much infrequent symptom of adult-onset MLD than previously reported; the most common symptoms were ataxia and behavioral abnormalities in 13 patients with adult-onset MLD. Furthermore, according to current literature, there is a correlation between genotype and phenotype. Baumann et al. (15) indicated that two clinical forms may be distinguished: one form is characterized by CNS motor signs and peripheral nerve neuropathy, while the other is characterized by psycho-cognitive abnormalities. Patients with psycho-cognitive dysfunction due to adult-onset MLD were found to have a specific compound heterozygous mutation, p.I179S, while patients with motor dysfunction appeared to have a homozygous p.P426L mutation (16). These two *ARSA* mutations comprise ~50% of all mutant alleles reported; the remaining mutations are rare. However, our patient had both these clinical forms, and the visual disturbance was more prominent. To the best of our knowledge, this has not been previously reported. Our patient had both motor and psycho-cognitive phenotypical forms, but the most common mutations of the adult phenotype, p.I179S and p.P426L, were absent. The p.I179S and p.P426L mutations were also not detected in two previous Chinese MLD cases (17, 18). It appears that *ARSA* genotypes in Chinese patients with MLD differ from those of patients in Western countries.

The adult-onset clinical symptoms are different from late-infantile and juvenile MLD. Late-infantile MLD is generally associated with weakness, inability to walk, mental retardation, increased muscle tone, or slurred speech. Furthermore, these patients show rapid and severe functional decline, while a similar and slower disease progression is shown in patients with the juvenile forms (19). The age at onset, duration of illness, and clinical progression are relevant variables in adult-onset MLD. Pure neurological deficits, hypotension, post-partum depression, and visual dysfunction are rare initial clinical presentations of adult-onset MLD with variable mutations (1, 3, 20, 21). Wright et al. (1) reported the case of two patients with hypotension as a cardinal symptom, but with intact adrenal function, differing from adrenoleukodystrophy, which shows clinical and biochemical features of adrenal failure. The urinary problem presented in these patients indicated autonomic dysfunction. The normal levels of the ARSA enzyme and a dominant inheritance in the family suggested this is an unusual example of MLD. Kumperscak et al. (20, 21) reported the cases of two female patients with MLD from different families who had post-partum depression as the main symptom. One patient carried a novel p.T279I mutation of the *ARSA* gene, which suggests that an *ARSA* gene mutation may lead to post-partum depression in young pregnant women.

Optic atrophy, usually apparent later in the disease course, is a consistent ophthalmological sign in MLD patients. Quigley et al. reported the cases of two adult patients with decreased vision (22). Abnormal optic nerve myelin metabolism and retrograde ganglion cell degeneration may be the primary pathologic ocular process. Decreased visual acuity and optic atrophy are well-known symptoms in MLD, independent of the age at onset. However, compared with infantile MLD patients, adult MLD patients have more extensive ganglion cell and optic atrophy, which may be a result of a longer survival into adulthood. Our patient had poor eyesight early in the disease course. The patient's unusual VEP suggested that the visual conduction from the optic nerve to the cerebral cortex was abnormal.

Adult-onset MLD predominantly affects the CNS; however, peripheral nerve involvement is a very important feature (15). Kappler et al. reported the cases of 14 patients with adult MLD, wherein most patients developed peripheral neuropathy due to reduced motor and/or sensory nerve conduction velocities (23). Moreover, a patient with isolated polyneuropathy could also be misdiagnosed as chronic inflammatory demyelinating polyradiculoneuropathy (4); however, a homozygous missense mutation, p.T286P, confirmed by molecular genetic analysis, could change the diagnosis to MLD. Genetic studies have been performed in other specific MLD cases with isolated polyneuropathy, which showed compound heterozygosity for the IVS2+1G→ A mutation and a newly identified missense mutation, p.T408I (2). These demonstrate that the genetic causes of adult MLD presenting as isolated polyneuropathy are complex. However, other cases with normal nerve conduction velocities in adult MLD have also been reported. Schneider et al. (24) and Marcão et al. (25) reported the novel p.F144L and p.F219V mutations of the *ARSA* gene in patients with adult-onset MLD, associated with psycho-cognitive impairment without clinical or electrophysiologic signs of PNS involvement. Cengiz et al. (26) revealed the cases of two familial patients with adult-onset MLD who presented with cognitive disturbances, but their nerve conduction velocities were normal. The combination of rapid cognitive decline and absent PNS involvement is unusual, which may be the result of different *ARSA* gene mutations causing isolated CNS involvement. Our patient had elevated protein levels and cell count in CSF. Electromyography was not done because of the brisk tendon reflexes and Babinski signs. Therefore, it could not be confirmed whether the peripheral nerves were involved in our patient.

Missense mutations are the most frequent type of defect found in the *ARSA* gene to date. As the biochemical characterization of the consequences of missense mutations is rather laborious, only about a dozen of the missense mutations have been examined at the biochemical level (27). The p.T393S mutation in the *ARSA* gene has been reported in the Human Gene Mutation Database (http://www.hgmd.cf.ac.uk/ac/index.php), but it has no clinical significance. We applied several variant prediction tools, including SIFT (http://provean.jcvi.org/), PolyPhen-2 (http://genetics.bwh.harvard.edu/pph2/), Mutation Taster (http://www.mutationtaster.org/), and CADD (https://cadd.gs.washington.edu/snv), to predict the functional impact of the novel missense variants. These variant prediction tools provided a damaging prediction of the p.P220L mutation caused by a substitution of proline-220 with leucine. The p.P426L is the most frequently reported mutation in adult patients with MLD (60%) (28). Hohlfeld et al. (29) demonstrated that the p.P426L mutation encodes a mutant ARSA enzyme with a low residual activity. Zlotogora et al. (30) reported a mutation, C>T at position 2119, causing proline-377 to be replaced with leucine (p.P377L). Biosynthesis studies performed with cells expressing the *ARSA* cDNA into which p.P377L mutation was introduced have shown that the half-life of the mutant enzyme was greatly reduced. The substitution of proline by leucine may have a major effect on the polypeptide structure and orientation. Therefore, it is likely responsible for the low residual ARSA activity. Genetic analysis revealed that our patient had a deletion in exon 4 of the *ARSA* gene, which was a novel mutation and not previously reported. Yang et al. found that 19 of all the reported deletion mutations were related to late-infantile MLD and that these mutations were distributed in exons 1, 2, 3, and 8. Furthermore, most of these mutations were frameshift mutations, indicating that the C- and N-termini of the ARSA enzyme play a key role in maintaining ARSA activity (31, 32). We speculated that the p.P220L mutation and a deletion of exon 4 in the *ARSA* gene may cause the low residual ARSA activity, which led to the faulty degradation of cerebroside sulfate and caused metabolic diseases. The cerebroside sulfate is a constituent of myelin and cellular membranes. The consequent abnormal storage of cerebroside sulfate can be seen in the white matter of the neuraxis and other organs. Therefore, the demyelination and axonal loss in white matter result in the abnormal visual conduction and binocular blurred vision in our patient. Because of the conditions, the sulfatase enzyme activity and urinary sulfatide excretion were not assessed in our patient.

A previous study showed that mutant genes containing several mutations lead to a great decrease in ARSA activity when compared to mutant genes with a single mutation. Therefore, each mutation may contribute to ARSA activity reduction and increased disease severity (33). Since the known mutation, p.T393S, in the *ARSA* gene has no clinical significance and the patient's family showed no clinical symptoms, the damaging p.P220L mutation and the deletion of exon 4 in the *ARSA* gene may have caused the patient to develop the disease by autosomal recessive inheritance.

In conclusion, we describe the rare case of a patient with adult-onset MLD whose initial prominent presentation was visual disturbance. Brain MRI findings, including white matter demyelination in MRI, led to an initial misdiagnosis of multiple sclerosis; however, bilateral symmetrical demyelination oriented us to consider the diagnosis of adult-onset MLD. Moreover, genetic analysis confirmed the diagnosis of MLD in our patient, showing that the novel p.P220L mutation and the deletion of exon 4 in the *ARSA* gene may have led to the pathogenesis of MLD. To the best of our knowledge, this is the first report of visual dysfunction as the first manifestation of adult-onset MLD, which may have some link to novel p.P220L mutation and the deletion of exon 4 in the *ARSA* gene. This was the main purpose of our report on this case. We are hoping to arouse clinicians' attention to these symptoms to avoid the misdiagnosis of MS and the missed diagnosis of MLD. The study will help us to better distinguish multiple sclerosis from MLD and further understand MLD.

## Data Availability Statement

The original contributions presented in the study are included in the article/supplementary material, further inquiries can be directed to the corresponding author/s.

## Ethics Statement

Written informed consent was obtained from the individual(s) for the publication of any potentially identifiable images or data included in this article.

## Author Contributions

XZ designed the project conception. LX, MZ, YW, ZW, JS, JZ, HY, ZY, and WY performed the literature search and analysis. LX wrote the manuscript with contribution from XZ. All authors read and approved the final manuscript.

## Conflict of Interest

The authors declare that the research was conducted in the absence of any commercial or financial relationships that could be construed as a potential conflict of interest.
